# Sacha Inchi (*Plukenetia volubilis*): Potential Bioactivity, Extraction Methods, and Microencapsulation Techniques

**DOI:** 10.3390/molecules30010160

**Published:** 2025-01-03

**Authors:** Sarah Gustia Redjeki, Alfa Fildzah Hulwana, Rizqa Nurul Aulia, Ira Maya, Anis Yohana Chaerunisaa, Sriwidodo Sriwidodo

**Affiliations:** 1 Undergraduate Program in Pharmacy, Faculty of Pharmacy, Universitas Padjadjaran, Sumedang 45363, Indonesia; sarah21006@mail.unpad.ac.id (S.G.R.); alfa21001@mail.unpad.ac.id (A.F.H.); 2 Master Program in Pharmacy, Faculty of Pharmacy, Universitas Padjadjaran, Sumedang 45363, Indonesia; rizqa16001@mail.unpad.ac.id (R.N.A.); ira16001@mail.unpad.ac.id (I.M.); 3 Department of Pharmaceutics and Pharmaceutical Technology, Faculty of Pharmacy, Universitas Padjadjaran, Sumedang 45363, Indonesia; anis.yohana.chaerunisaa@unpad.ac.id

**Keywords:** sacha inchi oil, oil recovery, alternative medicine, oxidative stability, microcapsule technologies

## Abstract

Sacha inchi (*Plukenetia volubilis* L.), an oilseed native to the Peruvian rainforest, has garnered attention for its valuable components and its potential applications in the food, pharmaceutical, and nutraceutical industries. Sacha inchi oil is rich in fatty acids, particularly omega-3, omega-6, and omega-9, along with antioxidants such as tocopherols, which collectively contribute to cardiovascular health, antioxidant, anti-inflammatory, antiproliferative, and neuroprotective effects. The susceptibility of the oil to oxidation poses significant challenges for both storage and processing, making it essential to employ microencapsulation technologies to preserve its integrity and extend shelf life. This paper aims to provide a review of the therapeutic potential, extraction methods, and microencapsulation strategies for enhancing the oil’s stability and bioavailability. Optimizing both extraction processes and encapsulation strategies would enhance the oil’s stability and bioavailability, enabling it to be more effectively utilized in functional foods and therapeutic applications across the nutraceutical and pharmaceutical fields.

## 1. Introduction

Sacha inchi (*Plukenetia volubilis* Linneo), a plant native to the Peruvian rainforest, is part of the Euphorbiaceae family. It is also known as Inca peanut, wild peanut, or mountain peanut. The name “sacha inchi” comes from the Quechua language which means “wild peanut”. This is because it was originally eaten as a nut by the Chanka and Mochica-Chimú tribes before the Inca. Characterized by its star-shaped capsules, sacha inchi fruit grows to a size of 3 to 5 cm. As it ripens, the fruit undergoes a color transformation from green to a blackish-brown shade. Each capsule contains edible seeds, which are dark brown and oval in shape [1,2]. Sacha inchi cultivation requires a warm climate, with temperatures ranging from 10 to 36 °C, annual rainfall of 850–1000 mm, and well-drained acidic soils, such as sandy or clay loams, at altitudes of 200–1500 m under high light exposure, primarily in moist-to-wet lowland forest environments [3,4,5]. The flowering of sacha inchi occurs within 3–5 months after planting, while the fruiting process is observed around 8–9 months after the initial planting [6]. Sacha inchi is a climbing plant with diverse applications, requiring a single planting while allowing for multiple harvests per year over a productive period of 15–20 years [4]. This plant is now grown in many places, including Asia (Thailand, China, Vietnam, Indonesia) and Central and South America. It is not only a good source of nutrition but also contributes to economic development [7,8,9]. Sacha inchi is renowned for its abundance of unsaturated fatty acids, particularly in its seed oil [10].

Sacha inchi oil (SIO) has promising applications in the food, pharmaceutical, and nutraceutical industries due to its valuable components. It is primarily composed of polyunsaturated fatty acids, comprising approximately 85–93% of its total fatty acid content [11]. Linolenic acid (omega-3) and linoleic acid (omega-6) are the predominant fatty acids present within this composition. These essential fatty acids are necessary for human health and can only be acquired through dietary intake. Additionally, oleic acid (omega-9) has been identified as a notable constituent of SIO. Omega-3, omega-6, and omega-9 are present in SIO in proportions of approximately 48%, 33.5%, and 9%, respectively [4]. It is noteworthy that the specific fatty acid composition of sacha inchi seeds can exhibit variation depending on factors such as cultivar, cultivation conditions, and the processing methods employed [2,12,13]. In general, the composition of oilseeds is shaped by the interplay between genetic and environmental factors. Environmental factors, such as temperature, water availability, and the length of the cultivation period, play a crucial role in influencing the composition of fatty acids in seed oil [14,15]. Processing methods, such as pre-extraction roasting, and extraction conditions play a significant role in determining the composition of oils. Research has demonstrated that different extraction techniques yield differences in fatty acid profiles [2].

In addition to having anti-inflammatory, antioxidant, and blood lipid-lowering properties, SIO also has potential as an anti-aging agent. A study by Musquera et al. (2012) [16] tested the anti-aging effects of SIO on cosmetic formulas. In this study, 30 women with signs of skin aging used cosmetic products containing SIO for four weeks. The results showed significant improvements in skin firmness, elasticity, and smoothness after regular use. These findings were supported by clinical and instrumental evaluations conducted using a skin elasticity meter.

Despite its various benefits, SIO contains unsaturated fatty acids, especially omega-3 acids, which have a high susceptibility to oxidation, so SIO was developed into a microencapsulated preparation. This microencapsulation of SIO will inhibit or delay the deterioration of unsaturated fatty acids from environmental factors, such as light, air, or moisture [17]. The unsaturated fatty acid content of oxidized SIO can also give rise to an unpleasant odor and taste. This leads to low acceptability for direct oral consumption. With microencapsulation technology, the unpleasant taste and odor of SIO can be well covered and can be accepted by the public for oral consumption [18].

The microencapsulation process involves enclosing small particles or droplets within a coating or embedding them in a matrix, either homogeneous or heterogeneous, to produce small capsules with advantageous properties. Through this technique, the encapsulated ingredients are fully surrounded by a coating material, thereby enhancing desirable characteristics or eliminating undesirable ones from the original substance [19]. Microencapsulation serves as a strategy to maintain the quality and enhance the physicochemical stability of components that are vulnerable to thermal or photodegradation, such as omega-3 fatty acid [20].

This study focuses on the biological activity, extraction and microencapsulation of SIO, three studies that have been individually researched but rarely explored together. By bridging these fields, this study aims to propose a comprehensive method for improving the efficiency and application of this natural resource.

## 2. Biological Activity and Recommended Dosing of Sacha Inchi Oil (SIO)

Sacha inchi, or *Plukenetia volubilis*, is a plant native to the Amazon region that is known for its abundance of essential fatty acids, especially omega-3, omega-6, and omega-9, as well as many antioxidants. SIO contains a diverse array of bioactive compounds that play a significant role in enhancing its nutritional value and therapeutic applications [3]. Essential fatty acids dominate the composition of SIO, with α-Linolenic and linoleic acid together contributing up to 82% to its content. Other bioactive components, such as tocopherols, carotenoids, polyphenols, and phytosterols, have also been identified in this oil [12,21,22,23,24].

Omega-3 and omega-6 fatty acids play a crucial role in reducing cardiovascular disease risk by lowering LDL cholesterol (omega-6) and triglycerides (omega-3), enhancing vascular health, and managing inflammation. They also contribute to brain development, cognitive health, and offer therapeutic benefits for conditions involving chronic inflammation, oxidative stress, and neurological disorders [12,25]. Omega-9 fatty acids, especially oleic acid, exhibit significant health-promoting properties that aid in wound healing and decrease eye inflammation by regulating inflammatory pathways. They also show potential anti-cancer properties, particularly in breast cancer, by limiting tumor cell growth and migration, though their efficacy varies across cancer types and require further research [26].

Tocopherols support cellular homeostasis, mitigate inflammation through antioxidant activity, and regulate signaling pathways, enzymatic functions, and gene expression [27]. Carotenoids contribute to reducing oxidative stress and minimizing the likelihood of developing chronic diseases, including cardiovascular, neurological, and metabolic disorders, as well as specific cancers [28]. Phytosterols demonstrate a range of health benefits such as lowering cholesterol levels and providing anticancer, anti-inflammatory, and immunomodulatory properties [29]. Similarly, phenolic compounds reduce cancer risk, enhance insulin sensitivity, reduce inflammation, and prevent liver-related and infectious diseases [30]. Table 1 presents the fatty acid content and bioactive compounds in SIO.

Due to its exceptional nutritional content, SIO has many pharmacological activities that are good for human health. Recent studies have shown that this oil has treatment potential for a variety of conditions, including cardiovascular health, anti-inflammatory, antioxidant, antiproliferative, and neuroprotection [7,31]. Table 2 is a summary of the therapeutic applications of SIO.

One of the pharmacological properties of SIO is that it benefits the heart and blood vessels. Clinical studies have shown that SIO contains Omega-3, specifically ⍺-linolenic acid (ALA), which lowers LDL cholesterol and triglyceride levels in the blood [32]. ALA is metabolized in the body into eicosapentaenoic acid (EPA), which is further converted into docosahexaenoic acid (DHA). These omega-3 fatty acids, EPA and DHA, are also recommended for cardiovascular health [33,34]. To determine the levels of fatty acid exposure, especially ALA, after a single administration of SIO, Gonzales et al. analyzed the plasma fatty acid levels in participants who consumed SIO versus sunflower oil. The study involved 18 healthy participants (9 males and 9 females, aged 20–55) consuming 10 or 15 mL of cold-pressed SIO. ALA peaked 4 h after intake, with plasma levels significantly higher in women (2.84 ± 0.36) than in men (0.94 ± 0.57). DHA levels were also elevated in both groups. This effect was not detected in those consuming sunflower oil [34]. In another study, they also found that the consumption of SIO has demonstrated efficacy in preventing dyslipidemia in healthy individuals. It was found that after 16 weeks of consuming 10 or 15 mL of SIO, participants experienced a significant decrease in total cholesterol and LDL cholesterol, along with an increase in HDL cholesterol [35].

In the study by Li et al., the activity of lipid dysmetabolism improvement was measured in obese rats. The findings show that 0.5–1.5 mL/kg SIO consumption for 8 weeks reduces gut microbiota dysbiosis and improves lipid metabolism in rats fed a high-fat diet (HFD). This process decreases de novo lipogenesis and promotes the β-oxidation of fatty acids, thereby correcting the imbalances in triglyceride (TG), glycerophospholipid, and sphingolipid metabolism. These improvements contribute to a reduction in low-grade inflammation, liver fat accumulation, and hyperlipidemia in mice on a high-fat diet [36]. The randomized controlled trial (RCT) study by Garmendia et al. on hypercholesterolemia patients also reported significant reductions in TC, LDL, non-HDL cholesterol, TGs, VLDL, and non-esterified fatty acids (NEFA). Simultaneously, HDL cholesterol levels rose significantly, achieving a 30% increase from baseline [37].

In addition to their well-documented benefits for cardiovascular health, polyunsaturated fatty acids (PUFAs) have been associated with neuroprotective properties [38,39]. Herrera-Calderon et al., therefore, examined the anticonvulsant activity of SIO against pentylenetetrazole (PTZ)-induced seizures. The experimental design consisted of five groups. Group I (control) received PTZ (80 mg/kg, s.c.), while Group II was given PTZ and diazepam (1 mg/kg, s.c.). Groups III, IV, and V were treated with PTZ along with single oral doses of SIO at 250, 500, and 1000 mg/kg, respectively. The oil was administered 30 min prior to PTZ-induced seizure induction. The study concluded that SIO demonstrated significant anticonvulsant effects at the highest tested doses, with effects closely resembling those of diazepam. The underlying mechanism is likely associated with its ability to reduce free radicals and enhance GABA levels in the brain [40].

SIO also has strong anti-inflammatory properties. The production of inflammatory mediators such as prostaglandins and leukotrienes can be reduced by the polyunsaturated fatty acids contained in this oil. Chronic inflammatory diseases such as arthritis and asthma can be treated with this anti-inflammatory effect. Eicosapentaenoic acid (EPA, ⍵-3) competes with arachidonic acid (⍵-6) for cyclooxygenase and lipoxygenase enzymes, reducing the production of prostaglandin E2 (PGE2), thromboxane A2 (TXA2), and leukotriene B4 (LTB4), which are involved in platelet aggregation, vasoconstriction, inflammation, and leukocyte adhesion [7].

Alayón et al. (2019) conducted a study to investigate the effects of adding SIO on postprandial inflammation and lipid profile due to high-fat intake. Two kinds of breakfasts were given to 20 metabolically healthy (MH) and 22 metabolically unhealthy (MU). The first breakfast was termed high saturated breakfast (HFM), and the second breakfast was HFM+S, which included an additional 15 mL of commercial SIO. The results showed that the addition of SIO induced a reduced postprandial serum total cholesterol (TC) and IL-6 in both groups and completely returned it to baseline in the MH group. However, it showed no significant effect on HDL-C and TG. This result may be due to the limited period of SIO consumption. SIO intake also resulted in a notable decrease in serum IL-6, especially within the MH group. In the MU group, SIO consumption reduced the increase in LPS and weakened the relationship between LPS and TG. This may suggest a potential inhibitory role of PUFAs in limiting LPS entry into circulation [41]. In another study, they also found that SIO consumption improved insulin sensitivity in the group with less favorable metabolic profiles and heightened glycemic responses to fat intake. Furthermore, increased expression of Sirtuin 1 (SIRT-1) was noted in individuals with higher initial triglyceride levels and glycemic responses. SIRT-1 expression in peripheral blood mononuclear cells (PBMC) was linked to enhanced postprandial insulin sensitivity [42].

Ambulay et al. reported in their study that the administration of 2.5 mL of SIO emulsion with different concentrations of ⍵-3 (0.5 g/day and 0.25 g/day) led to reduced levels of LDL-C, TC, and TG, while simultaneously increasing HDL-C levels. A reduction in malondialdehyde (MDA) and advanced oxidation protein products (AOPPs), along with an increase in catalase activity, was observed, indicating antioxidant effects. Additionally, SIO emulsion reduced inflammation in obese rats, as indicated by the decreased levels of pro-inflammatory cytokines IL-6 and TNF-⍺ [43]. Another study conducted by Suthiphasil et al. demonstrated the anti-inflammatory and antibacterial activities of SIO. The combination of 6 mL of SIO after breakfast, accompanied by a 250 mg erythromycin tablet before meals, four times a day for 8 weeks long, was significant for reducing acne severity and total acne count [44].

SIO also contains tocopherols, phenols, and carotenoids, providing antioxidant properties that aid in preventing oxidation [3,45]. The total tocopherol content in *P. volubilis* L. for the shell and seeds exceeds that of other oilseed crops, such as rye. Recognized as natural antioxidants, tocopherols prevent lipid oxidation by stabilizing hydroperoxyl and free radicals. Besides enhancing oil stability, they are crucial for human health, associated with delayed aging, lower cardiovascular disease risk, and cancer regression in cell cultures [3,46].

As of now, there is limited information regarding the association between sacha inchi or its derivatives and the prevention of neoplasia or tumors [7]. Despite this, Schiessel et al. produced noteworthy findings regarding the effects of a sacha inchi fatty acid supplement, which primarily consists of ALA, in the context of antineoplastic control. This was demonstrated by a reduction in tumor mass and Walker 256 cell proliferation by up to 2.3-fold and 3-fold, respectively, in a murine model of breast cancer. These findings align with data obtained for a fish oil diet and are accompanied by decreases of 65%, 62,5%, and 50% in plasma concentrations of TNF-⍺, IL-6, and triacylglycerides [47]. Centurion-Rodriguez et al. assessed the potential protective effects of SIO on colon cancer (CC) induced with 1,2-dimethylhydrazine (DMH) in Holtzman rats. The study was conducted by randomly assigning the rats into four groups: a positive control group receiving DMH (C1), a negative control group treated with SIO at 150 µL/kg/day (C2), and two experimental groups treated with both DMH and SIO at 150 µL/kg/day (E1) or 300 µL/kg/day (E2). DMH was administered for 8 weeks, with the total duration of induction lasting 22 weeks. The preventive effect was assessed by comparing the proportion of rats without lesions in the DMH-exposed groups. In this study, a 12.5% higher proportion of rats in the E1 group were protected from tumor induction, showing no injury, compared to the control group. Despite this, no statistically significant differences were observed, preventing a definitive link between SIO consumption and the prevention of neoplastic lesion formation [48].

**Table 2:** SIO therapeutic Applications.

Activity	SI Preparation	Dose	Experimental Models	Results	Reference
Antidyslipidemic	SIO suspension	5 or 10 mL for 4 months	Hypercholesterolemia patients aged 35–75	Significantly reduced the concentrations of TC, LDL, non-HDL cholesterol, triglycerides, VLDL, and NEFA. In contrast, HDL cholesterol levels rose significantly, reaching 30% above the initial measurement.	[37]
Antidyslipidemic	SIO	10 or 15 mL	18 healthy volunteers (9 males and 9 females) aged 20–55 years	Significant increase in plasma ALA 4 h after intake, (2.84 ± 0.36) mg/mL in women and (0.94 ± 0.57) mg/mL in men. As well as increase in DHA, (2.60 ± 0.84) mg/mL in women and (1.00 ± 0.38) mg/mL in men.	[34]
Antidyslipidemic	SIO	10 or 15 mL for 16 weeks	Non-vegetarian men and women aged 25–55 years, BMI ranging from 20 to 35 kg/m^2^, no history of hyperlipidemia or conditions that could influence lipid metabolism, not taking fatty acid, lipid, antioxidant supplements or medications; 18 healthy volunteers (9 males and 9 females) aged 20–55 years	At month-4, serum HDL-C increased, serum TC and LDL-C decreased, and arterial blood pressures reduced	[35]
Antiproliferative and antitumoral	SIO	1 g/kg for 4 weeks	Walker 256 tumor-bearing rats	Reduced tumor mass and proliferation of Walker 256 tumor cells ex vivo, increased lipoperoxidation in the tumor tissues, and reduced plasma levels of glycemia, triglycerides, and inflammatory cytokines	[47]
Antidyslipidemic and antioxidant	SIO	10% of the whole diet for 21 days	Male Wistar rats	Reduced ⍵-6/⍵-3 LCPUFAs from (9.24 ± 0.6) to (0.29 ± 0.03) g per 100 g of fatty acid methyl esters (FAME), and reduced expression and activity of Δ-5 and Δ-6 desaturases	[49]
Antiproliferative	SIO	150 and 300 µL/kg/day	Holtzman male albino rats	Cancerous lesions were present in two samples from group C_1_ (exposed to only DMH), one sample from group E_1_ (exposed to DMH with SIO 150 µL/kg/day), and two samples from group E_2_ (exposed to DMH with SIO 300 µL/kg/day). Group C_2_ (exposed to SIO 150 µL/kg/day) showed no intestinal lesions. The proportions of specimens without injury were 75% for group C_1_, 87.5% for group E_1_, and 75% for group E_2_, with no significant differences observed.	[48]
Antibacterial and anti-inflammation	SIO	6 mL for 8 weeks	32 volunteers with acne global severity 1–4	The combination of SIO extraction and oral erythromycin significantly reduced acne severity and total acne lesions	[44]
Antidyslipidemic and antihyperglycemic	SIO	15 mL	42 men aged 29–64, non-smokers, engaged in little physical activity	Increased insulin sensitivity, reduced postprandial hyperglycemia, and increased expression of SIRT-1 were noted in individuals with higher initial triglyceride levels and glycemic responses	[42]
Antidyslipidemic and anti-inflammation	SIO	15 mL	20 metabolically healthy (MH) and 22 metabolically unhealthy (MU) individuals given a high-fat meal	Reduced postprandial TC and IL-6 in both groups and the reverse in the MH group, no effect on HDL-C and TAG, and reduced LPS along with a suppression of the correlation between LPS and TAG in the MU group.	[41]
Antidyslipidemic	SIO	0.5–1.5 mL/kg for 8 weeks	High-fat diet (HFD)-fed rats	Reduced TC, TG, and LDL-C restored gut microbiota balance and enhanced bile acid profiles.	[36]
Neuroprotective	SIO	250, 500, 1000 mg/kg	Male Balb/C albino mice	The administration of SIO at its highest tested dose of 1000 mg/kg in the experimental group yielded better outcomes against PTZ-induced seizures, including decreased severity, reduced frequency, and shorter duration.	[40]
Antidyslipidemic, antioxidant and anti-inflammation	SIO emulsion	2.5 mL with different ⍵-3 contents (0.5 g/day and 0.25 g/day) for 8 weeks	Obese rats	Reduced LDL-C, TC, and TGs and increased HDL-C. Reduced MDA and AOPP and increased catalase activity. Reduced levels of IL-6 and TNF-⍺.	[43]
Anti-inflammation	SIO	2 × 530 mg softgel	Non-vegetarian adults aged 30 or older, with a BMI ranging from >20 to <35 kg/m^2^	Reduced the Systolic Blood Pressure (SBP) of the participants.	[50]
Anti-aging	SIO	Applied twice daily on the face and forearm area, with gentle massage until fully absorbed.	Thirty women aged between 26 and 65 years old with a certain degree of photo-aging were selected.	A 28-day treatment with TM001, TM003 and TM004 creams improved the condition of the volunteers’ facial skin. According to clinical evaluation, the creams improved the brightness and smoothness of the volunteers’ facial skin, while instrumental evaluation showed an increase in skin firmness and elasticity.	[16]
Anti-aging	SIO	30 and 15 mg/mL	Normal human fibroblasts (NHFs)	Sacha inchi was proven to be a promising plant for nutraceutical products, being rich in α-linolenic and linoleic acids, fatty acids that have health benefits.	[51]
Moiturizing	SIO	Applied on the left or right lower leg of the subjects for 14 days followed by application discontinuation for 2 days.	Skin tissues from surgery to remove excess skin from females in the age range of 50–65 years old were collected using 6 mm, full-thickness punch biopsies.	The oil was mild to the skin and had a beneficial effect on dry skin. Its moisturizing effect was comparable to that of olive oil.	[52]

Its important fatty acid content, especially omega-3, omega-6, and omega-9, which have antidyslipidemic and anti-inflammatory functions, makes SIO very popular. SIO contains alpha-linolenic acid (ALA), which functions as an antidyslipidemic, lowering blood levels of triglycerides and LDL cholesterol while also increasing HDL cholesterol levels. Both of these actions benefit heart health [34,35]. The ability of unsaturated fatty acids to stop the production of inflammatory mediators such as prostaglandins and leukotrienes is the reason for the anti-inflammatory properties of this oil [7]. The natural antioxidant content in SIO enhances this anti-inflammatory effect. This content protects body tissues from oxidative damage and oxidative stress, which aggravate inflammation [53].

As it is known that SIO is rich in omega-3, omega-6, and omega-9 fatty acids, SIO offers an optimal fatty acid profile for heart and brain health, making it suitable as a base for various food preparations. The distinctive texture and flavor of SIO make it suitable for use as a substitute for regular cooking oil, or as an addition to salad dressings and stir fry dishes, just like olive oil. SIO products have been sold widely and one that has been circulating in the Indonesian market is Svarga, from the collaboration between Quilla and the Faculty of Pharmacy, Padjadjaran University. However, people still rarely hear about this product and the great benefits of SIO. Therefore, more promotion is needed to increase interest in SIO products.

SIO is also still difficult to commercialize, not only as a food ingredient but also in other applications such as microencapsulation or even in cosmetics and pharmaceuticals. Some of the barriers to commercialization of SIO products include the following:

- Production Cost: The high production cost of SIO is due to the need for specialized equipment and advanced technology for the extraction and microencapsulation processes, as well as fluctuations in raw material prices.
- Production Limitations: The limited supply of sacha inchi seeds from certain regions and lack of large-scale production infrastructure are major constraints in meeting global market demand.
- Consumer Awareness: The lack of consumer awareness of the unique benefits of SIO over other vegetable oils, such as olive oil, necessitates more intensive promotional and educational efforts.
- Health and Safety Issues: The potential allergenicity and anti-nutritional compounds in SIO require special attention in the processing process to ensure product safety.
- Market Competition: Intense competition in the edible oil market, with the dominance of products such as olive oil and coconut oil, makes the penetration of SIO a challenge.

## 3. Extraction of Sacha Inchi Oil

The quality and bioactivity of SIO are determined by both upstream and downstream phases of production. In the upstream phase, the cultivation of sacha inchi plants is influenced by environmental and agricultural factors such as soil conditions, weather, and farming practices, all of which affect seed composition and oil yield [5,14]. Post-harvest operations, including drying and storage, also play a pivotal role in preventing oxidative deterioration and ensuring raw material quality [54,55].

Following these initial steps, downstream processes focus on the extraction and refinement of oil. A range of techniques have been explored for the extraction of natural oils, with variable yields depending on the specific method utilized [56]. Common techniques for extracting oil from plant seeds include traditional methods such as mechanical pressing, cold pressing, Soxhlet extraction, and shaking extraction (SE), as well as eco-friendly approaches like ultrasound-assisted extraction (UAE), microwave-assisted extraction (MAE), subcritical extraction (SubE), and supercritical fluid extraction [57]. SIO can be obtained from the sacha inchi seeds through several extraction techniques, such as cold pressing, conventional solvent extraction with polar and non-polar solvents, and enzymatic extraction [13]. The choice of extraction method has a significant impact on both the yield and the composition of bioactive compounds in SIO. Due to the oil’s rich content of valuable compounds and its numerous health benefits, many studies have concentrated on enhancing oil yield by exploring various extraction techniques [56,58]. Understanding the optimal extraction techniques is crucial for maximizing the benefits of SIO in industrial applications. A comparative analysis of the SIO yield produced by different extraction techniques is shown in Table 3.

The conventional production of SIO comprises a sequence of steps, beginning with the selection of sacha inchi seeds, followed by dehulling, cleaning, pressing, filtration, and final bottling. Dehulling is an advisable step in the processing of oilseeds [59,60]. A study by Gutiérrez et al. revealed that the oil extraction yield from whole sacha inchi seeds was substantially lower than that from dehulled seeds, likely due to the low lipid content of the seed shell, which accounts for approximately 35% of the total seed weight [61].

Among the different extraction methods, mechanical extraction stands out for its ease of setup, low investment and operational costs, lack of toxic solvents, and its effectiveness in achieving high oil yields [58,62]. Mechanical oil extraction employs either hydraulic presses or motor-driven screw presses [63]. The process involves placing the seeds between barriers, where compression reduces the space around the seeds, forcing the oil to be released [64,65]. Cold press extraction is a mechanical technique that is more energy-efficient than many other oil extraction methods and is environmentally sustainable. It is widely utilized for extracting oil from various sources, particularly oilseeds, and is considered eco-friendly as it does not involve the use of solvents. Furthermore, cold pressing avoids the use of heat or chemical processes to maintain the seeds’ nutritional value [66,67]. However, pressing methods have a relatively low extraction efficiency, leaving a significant amount of oil in the press cake, which is subsequently extracted using solvents like hexane [68].

Garcia et al. employed the cold pressing method using a filter press with 75 rpm and 240 Watt of power. The dehulled seeds were individually fed into the press, with an approximate interval of 5 s between each seed, then filtered before storage. The duration of the pressing and filtering process is approximately one hour, during which 120 mL of oil is directly obtained [67]. Gutiérrez et al. employed a similar method, where dehulled and whole sacha inchi seeds (1 kg each) were placed in separate filtration sleeves and pressed twice at 200 bar using a 15.24 cm diameter piston cylinder press. The pressing temperature remained below 30 °C throughout the process. The extraction yield from dehulled seeds was significantly higher (40.15 ± 2.50% *w*/*w*) than that from whole seeds (27.20 ± 1.90% *w*/*w*) [61].

To compare oil yield between different roasting temperatures, Cisneros et al. conducted a study by employing the pressing method to extract SIO. The kernels were roasted using a commercial gas-fired roaster, with each batch being approximately 4.5 kg. Three roasting conditions were tested: lightly roasted (75–81 °C for 9 min), medium roasted (83–86 °C for 10 min), and highly roasted (99–102 °C for 10 min). After roasting, the kernels were cooled using the roaster’s integrated fan. The unroasted sample and the three roasted variants were pressed with a temperature of 100–110 °C. The yield of non-clarified oil exhibited a progressive increase with higher roasting temperatures, achieving weights of approximately 42%, 49%, and 65% for lightly, medium, and highly roasted samples, respectively [55].

One of the approaches used in cold-pressed oil production is extraction via mechanical screw presses. Screw pressing offers several advantages, such as yielding high-quality oil with bioactive compounds, the potential use of the residual cake, and lower initial investment and operational expenses [69]. Muangrat et al. conducted research to assess the effects of operational parameters, including drying temperature (60, 75, 90 °C) and press head temperature (60, 75, 90 °C), on SIO extraction by utilizing a single screw press machine. The combined conditions of a feeding rate of 1.5 kg/h, a drying temperature of 90 °C, and a press head temperature of 90 °C resulted in a maximum extraction yield of 40.63% of SIO [62]. Mai et al. also employed screw pressing to obtain SIO. The extraction conditions were not directly reported, but the procedure yielded 47.5% oil [45]. This result is not significantly different from the oil yield achieved through screw pressing, as documented by Muangrat et al.

Hydraulic pressing is another mechanical extraction method that provides oil yields comparable to screw press extraction and higher quality oil than solvent extraction. However, it produces a lower oil yield than solvent extraction [58,70]. In hydraulic presses, pressure is generated by hydraulic equipment, which utilizes higher pressure than screw presses and can be operated either manually or with power [65]. A study by Chasquibol et al. reported the use of hydraulic pressing of *P. huayllabambana* L. seeds to obtain SIO. The procedure was conducted by applying pressure to the seeds using a piston driven by a hydraulic system, assisted by a 1-horsepower (HP) motor. Compared to *P. volubilis*, which is the most commonly used cultivar for oil extraction and commercialization, *P. huayllabambana* is nearly twice as large and heavy, yielding an oil content of 30.3 to 41.25% after pressing and filtration of the shelled seeds [71].

Kong et al. reported a study to determine the impact of pressure and pressing time on SIO yields [58]. A hydraulic cold-press extraction of the dehulled seeds was carried out at a fixed temperature of 25 ± 1 °C, with pressures ranging from 30 to 50 MPa and pressing times varying from 10 to 30 min. The study revealed that both pressure and pressing time significantly affected the yields of SIO, with the optimal oil recovery of 86.31% achieved at 50 MPa over 30 min. The oil yield obtained in this study was similar to the findings of Muangrat et al. regarding screw press extraction and Chasquibol et al. concerning hydraulic pressing [58]. Hidalgo et al. also implemented mechanical extraction through the use of extrusion. The extraction, carried out at room temperature with 1000 g of sacha inchi seeds, produced 26.92% oil. This yield was considered low, likely due to the absence of elevated temperatures during extraction [72].

Another method for extracting vegetable oils near room temperature is supercritical fluid extraction (SFE), often referred to as supercritical carbon dioxide extraction. SFE extracts substances from a matrix using supercritical fluids. A substance is classified as a supercritical fluid when it is held at pressures and temperatures beyond its critical points. These fluids exhibit molecular permeability and viscosity similar to gases, allowing for easy flow. Additionally, their low surface tension enhances mass transfer by increasing the contact area between phases during separation processes [73,74]. The use of low-to-moderate temperatures helps prevent unwanted reactions and product degradation. A primary advantage of this method is its suitability for extracting heat-sensitive compounds without the use of toxic solvents [13,64]. However, SFE could be considered too costly due to its high investment costs [75]. Additionally, SFE requires the careful optimization of various process parameters, such as temperature, pressure, flow rate, and solvent type, to achieve optimal extraction yields and product quality. This optimization process can be both time-consuming and resource-intensive [76]. Subtle changes in temperature and pressure can significantly impact the extraction yield and selectivity. Therefore, the precise control of these parameters is essential to maintain consistent product quality [77].

In their study, Zanqui et al. selected n-propane as the solvent for SFE due to its faster performance compared to CO_2_, attributed to its higher lipid solubility. The solvent n-propane was pressurized using a syringe pump, with temperature regulated by a thermostatic bath set at 10 °C. A flow of 1 mL/min was maintained for 1 h, regulated by an expansion valve at 80 °C. Temperature and pressure variations were also applied. The combination of a temperature of 60 °C and a pressure of 12 MPa resulted in the highest oil yield of 29.7 ± 0.5% [53].

Triana-Maldonado et al. (2017) also obtained SIO through SFE, employing CO_2_ as solvent. The extraction process involved a 200 g sample placed in a 1 L tank, operated at a temperature of 60 °C, with pressures between 400 and 500 bar, a CO_2_ flow rate varying from 40 to 80 g/min, and an extraction time between 120 and 180 min. The optimal oil extraction yield of 23.48 g of oil/100 g of samples was attained at a temperature of 60 °C, a pressure of 500 bar, a CO_2_ flow rate of 40 g/min, and an extraction duration of 180 min [78].

A similar approach was employed by Jitpinit et al. The extraction of SIO was carried out in a 100 mL extraction vessel. CO_2_ from an external cylinder was pressurized and liquefied using a high-pressure pump and a coolant bath attached to the pump head. The CO_2_ was then heated in a CO_2_ heater to reach supercritical conditions before entering the vessel, where 70 g of ground seed kernels was loaded. To evaluate the impact of temperature and pressure on the extraction of SIO using supercritical CO_2_, the process was carried out at temperatures of 40, 50, and 60 °C, and pressures of 300 and 400 bar. The CO_2_ flow rate remained constant at 1.5 L/min, with extraction continuing for up to 6 h. A maximum oil yield of 38.1% was achieved during supercritical CO_2_ extraction conducted at 400 bar and 60 °C, and optimum extraction time of 3 h [13].

Another extraction method that might be preferred is the solvent extraction method due to its cost-effectiveness and ease of use. This method relies on the solvent’s capacity to dissolve and extract oils from seeds. Thus, the solvent must effectively solubilize the oil to ensure efficient extraction. The primary drawbacks of solvent extraction are associated with the use of organic solvents, which can contribute to environmental pollution and pose health risks [79]. Moreover, residual solvent can negatively impact the quality, safety, and flavor of the final product. However, the complete elimination of solvent from the extracted oil can be difficult to achieve [80]. The solvent extraction process comprises three primary stages: cleaning and conditioning of the oilseeds, the extraction of oil, and the separation of the miscella [64]. Bueno-Borges et al. reported a study that utilized solvent extraction to obtain SIO. Around 5 g of ground seeds was placed in stoppered Erlenmeyer flasks and extracted with 30 mL of n-hexane under agitation for 4 h at room temperature. The mixture was then filtered and concentrated via a rotary evaporator at 30 °C, yielding around 1.9 g of oil [81]. Cordero-Clavijo et al. adopted a similar approach, which is extracting SIO by the solvent extraction method. In summary, SI seeds were ground and mixed with n-hexane at a 1:2 weight-to-volume ratio in amber glass bottles. The solution was incubated at 150 rpm and 30 °C for one hour. After a 30 min settling period, the supernatant (n-hexane and SIO) was filtered using Büchner funnels with Whatman No. 1 filter paper. The n-hexane was then removed from the SIO through rotary evaporation under vacuum at 50 °C, resulting in 97.22 ± 0.12% of oil [82].

The use of aqueous enzymatic extraction as an eco-friendly oil extraction method has become increasingly popular. Enzymes hydrolyze cell wall components, promoting the release of oil which can be effortlessly separated from the water phase, thereby making purification easier [83,84,85]. Nevertheless, aqueous enzymatic extraction has certain drawbacks, such as extended processing times, the potential for hydrolysis and oxidation of compounds, relatively high costs associated with enzymes or enzyme preparations, and the need for rapid processing of the unstable aqueous phase [86]. A study reported by Nguyen et al. demonstrated the extraction of SIO with aqueous enzymatic extraction. For this process, 2 g of seed powder was incubated with 1% (*w*/*w*) of various enzymes (chamzyme FP, flavourzyme, protamex, bromelain, papain, cellulase A, or a 1:1 blend of papain and cellulase A) in 10 mL of water at 40 °C for 4 h with stirring (6000 rpm). The mixtures were then centrifuged at 4000× *g* for 30 min, and the oil was collected, dried with anhydrous sodium sulfate, and heated at 105 °C for 3 h. Papain showed the highest efficacy for oil extraction, yielding 24.67% oil, outperforming the other enzymes tested [85].

**Table 3:** Different extraction methods for SIO.

Source	Extraction Method	Extraction Solvent	Extraction Conditions	Yield	Reference
*P. huayllabambana seeds*	Hydraulic cold pressing	-	Hydraulic piston with a 1 HP motor	30.3–41.25% of oil quantity	[71]
*P. volubilis seeds*	Pressing	-	Press temperature of 100–110 °C	65% of oil quantity	[55]
*P. volubilis seeds*	Supercritical fluid extraction (SFE)	n-propane	Temperature of 60 °C, pressure of 12 MPa, n-propane flow duration of 60 min, and extraction duration of 1 h	29.7 ± 0.5% of oil quantity	[53]
*P. volubilis seeds*	Supercritical fluid extraction (SFE)	Carbon dioxide	Temperature of 60 °C, pressure of 500 bar, CO_2_ flow rate of 40 g/min, and extraction duration of 180 min	23.48 g/100 g of samples	[78]
*P. volubilis seeds*	Screw pressing	-	Feeding rate of 1.5 kg/h, drying temperature of 90 °C, and press head temperature of 90 °C	40.63% of oil quantity	[62]
*P. volubilis seeds*	Solvent extraction	n-hexane	Temperature of 25 °C, extraction duration for 4 h and solvent volume of 30 mL	1.9 g of oil	[81]
*P. volubilis seeds*	Extrusion method	-	Temperature of 25 °C	26.92% of oil quantity	[72]
*P. volubilis seeds*	Cold pressing	-	Filter press with 75 rpm and 240 W of power	17.46% (120 mL) of oil quantity	[67]
*P. volubilis seeds*	Cold pressing	-	Piston cylinder press with pressure of 200 bar and temperature kept below 30 °C	40.15 ± 2.50% of oil quantity	[61]
*P. volubilis seeds*	Screw pressing	-	-	47.5% of oil quantity	[45]
*P. volubilis seeds*	Aqueous enzymatic extraction	Water	Mixture of seed powder with 1% *w*/*w* enzyme, 10 mL of solvent, temperature of 40 °C, extraction duration of 4 h, and stirring speed of 600 rpm	24.67% of oil quantity	[85]
*P. volubilis seeds*	Supercritical fluid extraction (SFE)	Carbon dioxide	Temperature of 60 °C, pressure of 400 bar, extraction duration of 3 h, and CO_2_ flow rate of 1.5 L/min	38.1% of oil quantity	[13]
*P. volubilis seeds*	Cold pressing	-	-	200 mL of oil	[14]
*P. volubilis seeds*	Hydraulic cold pressing	-	Temperature of 25 ± 1 °C, pressure of 50 MPa, extraction duration of 30 min	86.31% of oil quantity	[58]
*P. volubilis seeds*	Cold pressing	-	-	50% (1000 mL) of oil quantity	[87]
*P. volubilis seeds*	Solvent extraction	n-hexane	1 to 2 ratio of solids to solvent, stirring speed of 150 rpm, temperature of 30 °C, and extraction duration of 1 h	97.22 ± 0.12% of oil quantity	[82]
*P. volubilis seeds*	Supercritical fluid extraction (SFE)	CO_2_ and aqueous ethanol as cosolvent	Pressure of 400 bar, temperature of 60 °C, CO_2_ flow rate 10 g/min, extraction duration of 1 h	99.07 ± 0.03% of oil quantity	

Based on the table above, it can be presumed that supercritical CO_2_ extraction is a promising alternative for extracting SIO from P. volubilis seeds. This method enables the extraction of heat-sensitive compounds without the use of toxic solvents with faster extraction processes, excellent selectivity (resulting in high-quality extracts), and enhanced extraction yields. Furthermore, this method avoids the use of harmful solvents, preventing the formation of toxic byproducts and ensuring that the final food product is free from chemical residues [88,89].

## 4. Microencapsulation of Sacha Inchi Oil

Following extraction, microencapsulation techniques are utilized to augment the stability and bioavailability of SIO. These encapsulation methods safeguard the oil from oxidative degradation and enable its integration into diverse delivery systems [90]. Recent trends in the food sector show a significant increase in consumer preference for products categorized as healthy and natural [91]. This reflects people’s growing awareness of the importance of a nutritious and sustainable diet for long-term health. In response to this change in consumer behavior, research into new methods for incorporating nutritional supplements and bioactive compounds into people’s daily diets is gaining momentum. This research aims to create innovations in food formulations that not only fulfill nutritional needs but also enhance overall health benefits, thereby assisting consumers in achieving a healthier and more balanced lifestyle.

Essential oils, antioxidants, polyphenols, minerals, vitamins, and other bioactive compounds are examples of a wide variety of bioactive compounds with very diverse chemical properties. Beyond the basic nutrients commonly found in foods, these compounds have great potential to provide significant health benefits. They have anti-inflammatory activity that can help reduce chronic inflammation, anti-diabetic activity that can help regulate blood glucose levels, anti-cancer activity that can help fight cancer cell growth, anti-viral activity that can fight viral infections, and anti-tumor activity that can help treat malignant tumors [92]. However, these bioactive compounds must be protected to maintain their biological properties during storage and further incorporation into food formulations as they are susceptible to external stimuli such as light, oxygen, and temperature.

As is known, microencapsulation is an excellent technology to modify preparations containing bioactive compounds, especially fatty acid content. Microencapsulation is also one option to improve the bioavailability and stability of SIO, which is rich in omega-3 and omega-6 fatty acids that have many health benefits, such as improving heart health, reducing inflammation, and supporting brain function. However, the high content of unsaturated fatty acids makes this oil highly susceptible to oxidation, which in turn can reduce its quality, stability, and health benefits. Microencapsulation is therefore an excellent way to protect SIO from oxidation due to exposure to oxygen, heat, and light. It also increases its shelf life and stability. Microencapsulation encapsulates the oil with a protective coating such as maltodextrin or gum arabic. This preserves the bioactive properties of the oil during storage and allows the body to control its release when consumed [18]. Studies show that this method effectively reduces the peroxide value and TBARS (thiobarbituric acid reactivated substance) which are key indicators of lipid oxidation. In addition, this method removes the unpleasant taste and odor from the oil, making it easier to consume orally [20]. The process involves encasing the oil in a protective coating, which results in microcapsules that protect the vulnerable parts against heat, light, and oxygen. When used in food, medicine, or cosmetics, this method improves delivery and maintains the functional properties of the oil during storage [93].

One of the most commonly used microencapsulation techniques for oils, including SIO, is spray drying. In this method, an oil-in-water emulsion is created by mixing the oil with a wall material such as a protein, carbohydrate, or gum, such as maltodextrin, whey protein, or gum arabic. The emulsion is then atomized into fine droplets and dried quickly using hot air, resulting in microcapsules with a stable matrix. Spray drying has been shown to effectively improve the stability of SIO, reduce oxidation and extend shelf life, while maintaining the beneficial fatty acid profile [17].

Emulsions are highly susceptible to the negative impact of external stimuli, this can lead to fatty acid oxidation and during storage, thus limiting their application in the food industry [94]. Thus, the microencapsulation of emulsions via the spray-drying method has emerged as a promising strategy to extend product shelf life [95]. However, researchers have optimized the use of stabilizers and wall materials in this formulation, improving the stability of the emulsion during the spray drying process. This is because the stabilizers in the interfacial layer of the emulsion can be disrupted during the spray-drying process, leading to unstable emulsions and demulsification under high temperatures [32].

Spray-dried microencapsulation methods generally use protective materials such as maltodextrin, gum arabic, and OSA starch to protect the SIO emulsion. The spray-drying process using the B-290 apparatus involves the optimization of parameters such as inlet and outlet temperatures and flow rate. Temperature variation allows customization of the process for different types of samples. Inlet temperatures of 140–150 °C and outlet temperatures of 70–90 °C are used for the rapid evaporation of water and maintaining the stability of sensitive samples [90,96]. The flow rate of the feed solution and air significantly affects the atomization and drying process, thus having a direct impact on the size and particle size distribution of the final product [17,97].

Coacervation is a microencapsulation technique that involves forming a thin film around oil droplets using biopolymers such as gelatin or alginate. This method is preferred over spray drying for several reasons:

- High encapsulation efficiency: almost all oils can be encapsulated well.
- Better protection: heat-sensitive materials such as SIO are better protected.
- More uniform particle size: the resulting capsules tend to be larger and more uniform.
- Controlled release: the content in the capsule can be released slowly [98,99,100].

However, coacervation also has a more complex process. Ingredients such as tannic acid, pectin, and ovalbumin are often used as additional coacervation agents. Calcium chloride (CaCl_2_) plays an important role as a cross-linker to form a stable microcapsule structure [101,102,103].

Freeze drying is a microencapsulation technique that freezes the material first before removing the water directly, resulting in stable microcapsules. This method is particularly suitable for sensitive ingredients such as probiotics as it preserves their original physical and nutritional properties. In addition, the use of binders such as maltodextrin can increase the strength and protection of the microcapsules [18,104].

Each microencapsulation method has unique challenges. Spray drying can cause nutrient degradation in SIO due to high temperature-induced oxidation, as well as producing less uniform particles due to the high viscosity of the oil. Freeze drying requires a large capital investment for equipment and energy, as well as a long process time, making it less economically efficient. Coacervation has high process complexity due to sensitivity to various variables, and often results in lower encapsulation efficiency for oils with physicochemical properties such as sacha inchi.

In the food industry, spray drying, as it is known, is the most common method for drying microcapsules; freeze-drying is also frequently used. However, the two different types of drying are used to process the product. Each method results in variations in microstructure and particle size because it is performed at different temperatures and times. Freeze drying, on the other hand, is ideal for drying thermosensitive compounds because it is processed continuously at low temperatures. In the study by Suwannasang et al. in 2022, the authors used zein as a wall material for microencapsulation [105].

The oxidative stability of SIO was improved after being modified in the form of microencapsulation by various methods such as spray drying, freeze drying, and coacervation. Specifically, these microcapsules exhibit lower moisture content, water activity, hygroscopicity, and shorter sol time. Thus, a longer shelf life stored under controlled conditions with lower oxidation levels of the oil is expected. This was confirmed by the NMR results conducted by the researcher. Studies have shown that microencapsulation significantly improves the oxidative stability of SIO as shown in Table 4. Studies using encapsulation techniques such as spray drying and coacervation reported decreased peroxide and TBARS (thiobarbituric acid reactive substances) values, indicators of lipid oxidation, compared to non-encapsulated oils. This demonstrates the efficacy of this method in protecting the polyunsaturated fatty acids of SIO, thus making it more suitable for various applications that require long-term stability.

Based on Table 4, it is found that the spray-drying method is better used for the microencapsulation of SIO. The spray-drying process is an excellent choice for processing SIO microencapsulation due to its high efficiency, relatively low operating costs, and ability to produce highly stable microencapsulations. In this method, SIO emulsion containing coating materials such as maltodextrin and gum arabic is sprayed in the form of fine droplets into hot air, resulting in encapsulated dry microparticles. The main advantage of spray drying over other methods such as freeze drying and coacervation is its ability to produce microencapsulations in a short time with longer shelf life and sufficient protection against oxidation of unsaturated fatty acids [17]. In addition, spray drying is easy to implement on an industrial scale and can effectively preserve the nutritional properties of oils without requiring excessive temperatures, making it suitable for food and pharmaceutical applications [105].

Research has also shown that the microencapsulation of SIO using spray drying exhibits lower peroxide levels and better oxidative stability than other methods [18].

Microencapsulation of SIO has great potential but has not been optimally utilized. Some of its potential uses include the following:

- Pharmaceutical industry: the combination of omega-3 fatty acids in SIO with other bioactive compounds can increase drug effectiveness and facilitate body absorption.
- Food industry: microencapsulated SIO can be used to create innovative functional food products, such as chocolate or chewing gum with customized fatty acid profiles.
- Cosmetics industry: Microencapsulated SIO can be an active ingredient in skincare products, especially for anti-aging products. Its combination with natural antioxidants can enhance the effectiveness of the product.

In essence, microencapsulation of SIO opens up great opportunities for the development of innovative products in various industries, ranging from pharmaceuticals to cosmetics.

## 5. Conclusions, Limitations, and Future Perspectives

SIO exhibits considerable potential for implementation in the food, pharmaceutical, and nutraceutical fields due to its composition of essential fatty acids, particularly omega-3 fatty acids, and bioactive compounds. Numerous studies have demonstrated SIO, in various preparations, shows therapeutic potential for a range of health conditions, including benefits for cardiovascular health, antioxidant effects, anti-inflammatory properties, antiproliferative, and neuroprotective activity. This implies that SIO could be an important element in dietary regimens intended to enhance overall health and target specific medical issues. A notable limitation of this review is the insufficient number of large-scale clinical trials focusing on SIO, with the current evidence predominantly based on preclinical and small-scale clinical studies. Although these findings provide foundational knowledge, they may not be entirely representative of broader population groups, emphasizing the importance of conducting large-scale randomized trials. Moreover, sacha inchi research tends to emphasize certain cultivars or locales, with plant composition variations driven by genetic and environmental factors posing challenges to the universal applicability of the findings.

While the health benefits of SIO are promising and research gaps remain, it is imperative to also focus on resolving the practical issues related to its industrial utilization. The stability of SIO is a significant concern throughout the processes of extraction, handling, and storage. Thus, various studies have been conducted to optimize extraction parameters to enhance the efficacy of SIO in industrial applications. Additionally, the development of advanced microencapsulation techniques has been actively explored in order to enhance its stability and bioavailability. Further research should prioritize clinical trials aimed at validating the health benefits of SIO, and exploring its synergistic interactions with other nutraceuticals may also uncover additional therapeutic advantages.

## Figures and Tables

**Table 1:** Composition of fatty acids and bioactive compounds in SIO.

Component	Composition	Reference
**Fatty acids**		[21]
(Palmitic) C_16:0_	3.95 ± 0.21%	
(Palmitoleic) C_16:1_	0.06 ± 0.01%	
(Margaric) C_17:0_	0.09 ± 0.01%	
(Heptadecenoic) C_17:1_	0.05 ± 0.01%	
(Stearic) C_18:0_	3.05 ± 0.15%	
(Oleic) C_18:1_	9.90 ± 0.35%	
(Linoleic) C_18:2_	34.42 ± 0.58%	
(α-Linolenic) C18:3	47.88 ± 0.44%	
(Arachidic) C_20:0_	0.32 ± 0.15%	
(Gadoleic) C_20:1_	0.28 ± 0.15%	
**Tocopherols**		
γ-tocopherol	1142 ± 4 mg/kg	[22]
δ-tocopherol	715 ± 6.7 mg/kg	
Total tocopherol	1856 mg/kg	
Total carotenoids	1.0 ± 0.0 mg/kg	
**Phytosterols**		
Campesterol	10.47%	[23]
Stigmasterol	20.27%	
β-Sitosterol	54.44%	
**Polyphenols**		
Secoiridoids	40.96%	[24]
Flavonoids	14.40%	
Lignans	19.69%	
Isocoumarin	10.56%	
Phenyl alcohols	14.39%	

**Table 4:** Different microencapsulation methods for SIO.

Microencapsulation Method	Coating Materials	Microencapsulation Conditions	Oxidative Stability	Reference
Coacervation	Ovalbumin and sodium alginate	OVA and SIO solutions were mixed in an oil-in-water (O/W) emulsion, then homogenized with AL solution. The emulsion was set at pH 3.8, cooled to 10 °C for complex coacervation, and centrifuged. The microcapsules formed were cross-linked with CaCl_2_ solution, then dried using lyophilization after freezing at −40 °C.	This method improved the thermal resistance of the isolated bio-polymers (OVA and AL). This was confirmed by DSC and encapsulation by complex coacervation. The oxidative state of SIO was not affected by this method (as confirmed by 1H NMR).	[98]
Freeze-dried	Ovalbumin, XG, and PEC	Emulsions were prepared with varying concentrations of Ova, XG, or Pec, homogenized with UltraTurrax at 18,000 rpm, sonicated for 12 min, and freeze-dried to form microencapsulated products.	Freeze-drying and emulsion systems protect the omega-3 s without damaging the contents, as evidenced by NMR analysis for 180 min. Omega-3 is protected in SIO-Ova-Pec and SIO-Ova-XG microcapsules.	[20]
Spray-dried	Maltodextrin and Sodium caseinate	Microencapsulation was carried out by the spray-drying method using a Mini Spray B-290 at an inlet temperature of 150–170 °C and outlet temperature of 120–130 °C, with a 90% suction rate and 10% pumping rate; then, the microcapsules were stored in a dryer for further characterization.	In particular, these microcapsules exhibit lower moisture levels, water activity, and hygroscopicity, and shorter sol time. Thus, a longer shelf life stored under controlled conditions with lower oil oxidation levels is expected.	[90]
Spray-dried	Gum arabic	A solution with GA and distilled water containing camu camu and mango peel extracts was mixed with SIO (18% total solids) and homogenized. The emulsion was dried using a spray dryer at 140 °C and 70 °C with a flow rate of 55 mL/min, and the dried powder was stored in airtight bags at −5 °C for analysis.	Oxidative stabilization of microcapsules: natural (188–198 °C) vs. commercial (174 °C) antioxidants for inchi sacha oil.	[17]
Coacervation	Ovalbumin, PEC, and tannic acid	The microencapsulation process was carried out through these main steps: O/W primary emulsion with SIO and OVA-TA, followed by complex coacervation with PEC solution at pH 3.5, slow cooling, centrifugation, and drying by lyophilization.	There were no oxidatively decayed SIO products seen during the microencapsulation procedure.	[106]
Spray-dried	Gum arabic and maltodextrin	The microencapsulation method was performed by mixing gum arabic, maltodextrin, and whey protein isolate as encapsulation materials together with SIPHO oil, then homogenized at 9000 rpm for 10 min. After that, the emulsion was dried using a spray dryer, and the resulting powder was stored for further analysis at 5 °C.	Better oxidative stability and shelf life than microcapsules with BHT at 200 ppm.	[107]
Freeze-dried	Zein and maltodextrin	The emulsion was prepared through the liquid–liquid dispersion process, zein stock solution was prepared by dissolving zein in aqueous ethanol, and surfactant stock solution was prepared with Tween 80 and lecithin in pH adjusted deionized water. The mixture of surfactant solution, zein solution, and SO was homogenized at low temperature; then, ethanol was evaporated before the samples were frozen and dried to produce FDSOM powders.	The oxidative stability of microencapsulated SO in freeze-dried powders is anticipated to last longer than 15 days because yogurt is kept in a refrigerator.	[18]
Spray-dried	OSA starch and maltodextrin	Microencapsulation was carried out by preparing emulsions of hydrated starch MD and OSA, then mixing with SO and homogenizing. The emulsion was dried using a spray dryer at 150 °C (inlet) and 90 °C (outlet), with a feed rate of 0.8 L/h and an air flow of 35 L/min.	The oxidative stability of microencapsulated SO in spray-dried powders is anticipated to last longer than 15 days because yogurt is kept in a refrigerator.	
Spray-dried	Gum arabic	Microcapsules were prepared from a mixture of phenolic camu camu and/or mango peel extracts with gum arabic and sacha inchi *P. huayllabambana* oil. Emulsions were made using distilled water containing camu camu and mango peel extracts. The drying process was carried out with inlet and outlet air temperatures of 140 °C and 70 °C, respectively, and an optimal flow rate of 55 mL/min.	The microcapsulated antioxidants camu-camu peel and mango peel proved to be better at protecting against oxidation than the synthetic antioxidant BHT.	[108]
Spray-dried	Gum arabic, maltodextrin, whey protein isolate, Hi-cap, Capsul Tapioca starch	Coating material solutions were prepared with MD, Capsul TA, Hi-cap, AG, and WPI according to specifications. Emulsions were made with a homogenizer at low temperature before being dried using a spray dryer at 150–80 °C with a certain flow rate. The resultant dried powder was stored at low temperature for subsequent analysis.	The encapsulation process transformed the oil with a loss of 6.2% of the 3-Ln major fatty acids. SIHO with ternary coating materials (AG + MD + WPI) resulted in a 3% loss, while SIVO with Capsul TA as wall material only 0.2–0.9 mgg 1 oil for TG-P. The largest losses were tocopherols (30–15% for SIHO and surface, 28–24% for SIVO).	[21]
Spray-dried	Gum arabic and maltodextrin	AG and MD were used as encapsulating agents according to the previous method. CP was dispersed in ultrapure water at 80 °C with a concentration of 0.6% for 3 h. Antioxidant extracts GOE and ACHPE were added to the formulation. The total solids were adjusted to 30%. SIO was added at a concentration of 33.33% of the total solids. A homogeneous emulsion was obtained with a Silverson homogenizer. Spray drying was performed at 140–70 °C and a flow rate of 55 mL/min.	Formulation 3 (CP + SIOPH + GOE (200 ppm) + GA) with orujo grape extract had the longest shelf life (4908 ± 184 h). Formulation 5 (PC + SIOPH + ACHPE (200 ppm) + GA) (4740 ± 59 h) with aji charapita pepper extract was also stable.	[109]
Spray-dried	Gum arabic	Feed solution was prepared from AG-Inu and distilled water, and stirred for 12 h. SIO and polysorbate 80 were added, homogenized, and dried into microparticles.	-	[110]
Spray-dried	Lycopodium clavatum spores (average diameter of 25 μm)	SIO was dissolved in ethanol (9% *v*/*v*, 2 mL) and stirred for 5 min. Microcapsules (150 mg) were resuspended in SIO solution and rotated for 5 min at 1000 rpm. The SIO/spore ratios were 0.25:1.00, 0.50:1.00, 0.75:1.00, and 1.00:1.00 with codenames SIO@spore-0.25, SIO@spore-0.50, SIO@spore-0.75, and SIO@spore-1.00, respectively. The original spores were processed similarly. The mixture was shaken horizontally at 250 rpm for 24 h; then, the microcapsule spores (SIO@spore) were dried and stored at 4 °C before use.	The encapsulated microcapsule spore maintained its same form after 336 h of UV exposure, exhibiting little damage or distortion, indicating that UV radiation had a limited influence on its shell structure.	[111]
Spray-dried	Gum arabic and maltodextrin	The spray drying process was carried out using a laboratory-scale Büchi B-290 spray dryer with a 0.7 mm diameter nozzle, an air flow rate of 31.5 m^3^/h, and an air pressure of 50 mbar. The microcapsules were collected at −15 °C.	The use of WPC slows down the oxidation of SIO lipids in microencapsulation, indicating better oxidative stability.	[112]

## Data Availability

All data are contained within the article.
